# Incidence and risk factors for postoperative lingual neuropraxia following airway instrumentation: A retrospective matched case-control study

**DOI:** 10.1371/journal.pone.0190589

**Published:** 2018-01-12

**Authors:** Yi-Kai Su, Jen-Hung Wang, Shiu-Ying Hsieh, Xiu-Zhu Liu, Chen-Fuh Lam, Shian-Che Huang

**Affiliations:** 1 Department of Anesthesiology, Buddhist Tzu Chi General Hospital and Tzu Chi University School of Medicine, Hualien, Taiwan; 2 Department of Medical Research, Tzu Chi General Hospital, Hualien, Taiwan; 3 Department of Anesthesiology, E-Da Hospital/E-Da Cancer Hospital/I-Shou University, Kaohsiung, Taiwan; Southeast University Zhongda Hospital, CHINA

## Abstract

**Background:**

Lingual nerve injury or neuropraxia is a rare but potentially serious perioperative complication following airway instrumentation during general anesthesia. This study explored the the incidence and perioperative risk factors for lingual nerve injury in patients receiving laryngeal mask (LMA) or endotracheal (ETGA) general anesthesia in a single center experience.

**Methods and results:**

All surgical patients in our hospital who received LMA or ETGA from 2009 to 2013 were included, and potential perioperative risk factors were compared. Matched controls were randomly selected (in 1:5 ratio) from the same database in non-case patients. A total of 36 patients in the records had reported experiencing tongue numbness after anesthesia in this study. Compared with the non-case surgical population (n = 54314), patients with tongue numbness were significantly younger (52.2±19.5 vs 42.0±14.5; P = 0.002) and reported lower ASA physical statuses (2.3±0.7 vs 1.6±0.6; P<0.001). Patient gender, anesthesia technique used, and airway device type (LMA or ETGA) did not differ significantly across the two groups. A significantly higher proportion of patients underwent operations of the head-and-neck region (38.9 vs 15.6%; P = 0.002) developed tongue numbness after anesthesia. Multivariate logistic regression analysis indicated that head-and-neck operations remained the most significant independent risk factor for postoperative lingual nerve injury (AOR 7.63; 95% CI 2.03–28.70).

**Conclusion:**

The overall incidence rate of postoperative lingual neuropraxy was 0.066% in patients receiving general anesthesia with airway device in place. Young and generally healthy patients receiving head-and-neck operation are at higher risk in developing postoperative lingual neuropraxy. Attention should be particularly exercised to reduce the pressure of endotracheal tube or laryngeal mask on the tongue during head-and-neck operation to avert the occurrence of postoperative lingual neuropraxy.

## Introduction

Lingual nerve injury or neuropraxia is a rare but potentially serious perioperative complication following airway manipulation [1], particularly in patients received supraglottic airway instrumentation [2] or endotracheal intubation [3] during general anesthesia. Lingual nerve injury commonly presents with unilateral or bilateral tongue numbness and altered taste perception [4]. Although symptoms of lingual neuropraxia often subside spontaneously after a few weeks [5,6], some patients may experience prolonged tongue numbness for up to six months [7].

The lingual nerve lies beneath the mucosa on the inner surface of the mandible below the roots of the third molar and and innervates the sensory and taste sensation of the anterior two thirds of the tongue [8]. It is vulnerable to compression and stretching by laryngeal mask airways (LMA), endotracheal tube general anesthesia (ETGA), and other devices situated on the base of the tongue and inner surface of the mandible close to the third molar [9]. Patients who received supraglottic airway instrumentation [2] or endotracheal intubation [3] during general anesthesia are particularly at risk. Case-series studies and case reports also suggest that patient-related (such as diabetes, peripheral vascular disease), anesthesia-related (such as the size and placement of airway devices, cuff pressure, and poor technique) and surgical-related (patient positioning, head rotation, prolonged operation time) risk factors might have contributed to the development of postoperative lingual nerve injury [9], but the exact incidence and risk factors for postanesthesia lingual neuropraxia are still undetermined [2,9]. Since prospective study design is underprivileged for the rare clinical events [10], we retrospectively analyzed all events of tongue numbness after airway instrumentation during general anesthesia from 2010–2013 in our hospital. The aim of this study was to determine the incidence and risk factors associated with the development of post-anesthesia lingual neuropraxia. Our long-term goal was to develop preventive strategies for intraoperative lingual nerve injuries due to airway instrumentation.

## Methods

### Clinical database and study design

This retrospective chart-review study was approved by the institutional review board (IRB, Approval number IRB106-22-B) of Hualien Buddhist Tzu Chi General Hospital Taiwan and the requirement for written informed consent was waived by the ethics committee. Our hospital is a tertiary teaching medical center supporting 346 surgical beds. From January 2010 to December 2013, all patients received anesthesia management for surgical or other medical interventions were visited within 24 hours after operation. Post-anesthesia lingual nerve injury (neuropraxia) was defined as the development of numbness on the anterior tongue with altered taste perception (dysgeusia) and/or speech articulation after anesthesia [9]. As all patients who complained of tongue numbness received ETGA or LMA (Table 1), matched controls were randomly chosen from surgical patients who received intraoperative airway instrumentation (LMA or ETGA) but didn't report abnormal sensory changes on the tongue during the same study period. Age, American Society of Anesthesiologists physical status (ASA PS), and gender were matched between case (numbness) and control (non-numbness) patients in a 1 to 5 ratio. These characteristic parameters were chosen as the matching variables as they are considered strong confounders [11]. Patients who only received regional anesthesia, dental, or orthognathic surgery and patients who were discharged from the hospital within 24 hours post-operative were excluded from the study.

**Table 1:** Characteristics of patients with postoperative lingual neuropraxy.

Characteristics	n or mean±SD
Age (years)	42.1±14.4
Gender (F:M)	21:15
ASA PS class (I:II:III:IV)	16:18:2:0
Types of airway device (ETGA:LMA)	18:18
Duration of anesthesia (min)	132.9±66.4
Position during operation	
Supine	30
Lithotomy	1
Lateral decubitus	5
Types of operation	
Head-and-neck surgery	14
Thyroid/parathyroid surgery	3
Tonsillectomy/uvulopalatopharyngoplasty	4
Tympanoplasty	2
Parotid surgery	1
Cervical spine surgery	1
Submandibular sialolithiasis excision	1
Functional endoscopic sinus surgery	1
Open reduction for zygomatic fracture	1
Non-head-and-neck surgery	22
Orthopedic surgery on limbs	11
Plastic surgery	5
Laparoscopic surgery	2
General surgery	2
Chest surgery	1
Urology surgery	1

ASA ASA PS: American Society of Anesthesiologists physical status; ETGA: endotracheal general anesthesia; LMA: laryngeal mask anesthesia).

### Statistical analysis

The incidence of post-anesthesia tongue numbness was calculated as numbers of cases divided by total number of in-hospital surgical patients who received LMA or ETGA management during the study period. The size of the airway device was defined as usual (endotracheal tube ID ≤7.0mm for female and ID ≤7.5mm for male; LMA ≤3# for female and ≤4# for male) or large (any device size larger than those defined as usual in female and male). Difficult intubation was defined as failure to establish a secure airway (i.e. endotracheal tube or LMA) after 3 attempts by a senior anesthetist. Clinical anesthesia experience (in years) of the intubation operator was defined as junior (<5 years) or senior (≥5 years). The values of continuous variables were compared using an independent two-sample t test, one-way ANOVA or Wilcoxon rank-sum test, as appropriate. Categorical variables were compared using chi-square or Fisher’s exact test. Conditional logistic regression model was adopted to evaluate the associated risk factors (patient demographic and clinical variables) and postoperative tongue numbness. Statistical significance was accepted at a level of P< 0.05. All statistical analyses were performed using SAS 9.4 (SAS Institute, Inc., Cary, North Carolina).

## Results

It was recorded that a total of 36 patients reported tongue numbness after anesthesia over the 4-year study period, resulting in an overall incidence rate of 0.066%. A summary of patient demographic and operation information can be found in Table 1 and additional data are provided in S1 Table. There were 14 (38.9%) patients received operation on the head-and-neck regions, and equal number of patients (18 cases each) underwent ETGA or LMA. Patients who reported tongue numbness after operations were significantly younger (52.2±19.5 vs 42.0±14.5; P = 0.002) and in lower ASA PS (2.3±0.7 vs 1.6±0.6; P< 0.001) (Table 2) compared to the non-case surgical population (n = 54330). Gender distribution and anesthetic techniques (ETGA or LMA) did not significantly differ between the case and non-case populations (Table 2).

**Table 2:** Characteristic analysis of postoperative lingual neuropraxy (LN) in at-risk patients.

Characteristics	LN n = 36	Non-LN n = 54314	P value
Age (years)	42.0±14.5	52.2±19.4	0.002*
Age group (years)			0.008*
≤30	10(27.8%)	8027(14.8%)	
30–50	15(41.7%)	15296(28.2%)	
50–70	9(25.0%)	20735(38.2%)	
>70	2(5.6%)	10256(18.9%)	
Gender			0.131
Male	15(41.7%)	29933(55.1%)	
Female	21(58.3%)	24381(44.9%)	
ASA PS	1.6±0.6	2.3±0.7	<0.001*
ASA PS			<0.001*
I-II	34(94.4%)	34468(63.5%)	
>III	2(5.6%)	19846(36.5%)	
Site of operation			<0.001*
Non-head-and-neck surgery	22(61.1%)	53226(98.0%)	
Head-and-neck surgery^†^	14(38.9%)	1088(2.0%)	
Types of anesthesia			0.739
ETGA	18(50.0%)	29212(53.8%)	
LMA	18(50.0%)	25102(46.2%)	

ASA PS: American Society of Anesthesiologists Physical Status; ETGA: endotracheal general anesthesia; LMA: laryngeal mask anesthesia.

^†^The types of head-and-neck surgery are described in Table 1. Data are presented as mean±SD or n (%).

*P< 0.05 is considered as statistically significant.

A total of 180 patients case-matched for age, gender, ASA PS, and anesthetic technique undergone were randomly selected from the non-case population in order to identify additional potential risk factors for postoperative tongue numbness. A comparison of patient characteristics (body mass index, BMI), surgery-related factors (regions of operation and patient positioning), and anesthesia-related factors (size of airway device, clinical experience of anesthetist for airway instrumentation, duration of anesthesia and volume of fluid administered) between patients who reported tongue numbness and those who did not are presented in Table 3. Patient characteristics and anesthesia-related factors were not found to be significantly different across the two groups. The differences between the two groups remained insignificant after adjusting for patients’ weighted BMI with the size of the airway device (BMI-to-device ratio). A significantly higher proportion of patients who reported postoperative tongue numbness had undergone surgeries in the head-and-neck regions (38.9 vs 15.6%, case vs matched controls; P = 0.002) (Table 3). Patient positioning during operations was also found to be significantly difference across the two groups (Table 3). Fewer patients were placed in lithotomy (2.8 vs 10.6%, case vs matched controls) and prone positions (0 vs 14.4%, case vs matched controls) in the case group. Hospital stay length was not affected by the occurrence of postoperative tongue numbness (Table 3).

**Table 3:** Characteristic analysis of postoperative lingual neuropraxy in cases and matched control patients.

Characteristics	Cases n = 36	Matched controls n = 180	P value
Age(year)	42.1±14.4	42.1±14.3	1.000
Age Group			1.000
≦30 y/o	10(27.8%)	50(27.8%)	
30–50 y/o	15(41.7%)	75(41.7%)	
50–70 y/o	9(25.0%)	45(25.0%)	
>70 y/o	2(5.6%)	10(5.6%)	
Gender			1.000
Male	15(41.7%)	75(41.7%)	
Female	21(58.3%)	105(58.3%)	
ASA PS			1.000
1	16(44.4%)	80(44.4%)	
2	18(50.0%)	90(50.0%)	
≥3	2(5.6%)	10(5.6%)	
Type of anesthesia			1.000
ETGA	18(50.0%)	90(50.0%)	
LMA	18(50.0%)	90(50.0%)	
Size of airway device^†^			0.448
Usual	15(41.7%)	62(34.4%)	
Large	21(58.3%)	118(65.6%)	
BMI	25.8±6.4	24.7±4.5	0.220
BMI Group			0.476
18.4–24.9	11(30.6%)	73(40.6%)	
<18.5	3(8.3%)	10(5.6%)	
>25	22(61.1%)	97(53.9%)	
Site of operation^§^			0.002*
Non-head-and-neck surgery	22(61.1%)	152(84.4%)	
Head-and-neck surgery	14(38.9%)	28(15.6%)	
Difficult intubation^‡^			0.604
No	34(94.4%)	175(97.2%)	
Yes	2(5.6%)	5(2.8%)	
Experience of anesthetist^¶^			0.836
Junior	9(25.0%)	48(26.7%)	
Senior	27(75.0%)	132(73.3%)	
Intraoperative positioning			0.010*
Supine	30(83.3%)	120(66.7%)	
Lateral decubitus	5(13.9%)	15(8.3%)	
Lithotomy	1(2.8%)	19(10.6%)	
Prone	0(0.0%)	26(14.4%)	
Anesthesia time (min)	132.9±66.4	133.8±103.3	0.963
Intraoperative fluid administrated (ml)^Ω^	600(587.5)	500(900.0)	0.359
Length of hospital stay (days)^Ω^	4.0(3.0)	4.0(5.8)	0.739

ASA PS: American Society of Anesthesiologists Physical Status; BMI: body mass index; ETGA: endotracheal general anesthesia; LMA: laryngeal mask anesthesia.

^†^Size of airway device was defined as usual (endotracheal tube ID ≤7.0mm for female and ID ≤7.5mm for male; LMA ≤3# for female and ≤4# for male) or large (any device size larger than those defined as usual in female and male).

^‡^Difficult intubation was defined as failure to establish a secure airway (i.e. endotracheal tube or LMA) after 3 attempts by a senior anesthetist.

^§^The types of head-and-neck surgery are described in Table 1.

^¶^Clinical anesthesia experience (in years) of operator for intubation was defined as junior (<5 years) or senior (≥5 years). Data are presented as mean±SD, median (interquartile range) ^Ω^ or n (%).

*P< 0.05 is considered as statistically significant.

After multivariate logistic regression analysis, operations on the head-and-neck regions were still significantly higher in patients with tongue numbness with an adjusted odd ratio (AOR) of 7.63 (95% CI 2.03–28.70) (Table 4). However, the effect of patient positioning during operations became insignificant after multivariate analysis (Table 4). As patient age and ASA PS were matched in the second stage analysis, multivariate analyses were performed to confirm the interactions between these two parameters and head-and-neck surgery in the entire study population. During the study period, 53248 patients received non-head-and-neck surgery and 1106 patients received head-and-neck surgery. The multivariate regression analysis indicated that head-and-neck surgery and lower ASA PS class (I-II) remained associated with a significantly increased risk of postoperative lingual neuropraxy, while no significant differences in incidence between the age groups were found (Table 5).

**Table 4:** Conditional logistic regression analysis of the risk factors associated with postoperative lingual neuropraxy (cases vs matched controls, n = 36 vs 180).

Characteristics		Case		Matched control		AOR	95% CI	P value
		n	%	n	%			
Body mass index								
18.5–24.9		11	30.6	73	40.6	Ref		
<18.4		3	8.3	10	5.6	2.47	0.50, 12.11	0.265
>25		22	61.1	97	53.9	1.37	0.58, 3.24	0.474
Site of operation^†^								
Non-head-and-neck surgery		22	61.1	152	84.4	Ref		
Head-and-neck surgery		14	38.9	28	15.6	7.63	2.03, 28.70	0.003*
Difficult intubation^‡^								
No		34	94.4	175	97.2	Ref		
Yes		2	5.6	5	2.8	1.27	0.14, 11.75	0.836
Experience of anesthetist^§^								
Junior		9	25.0	48	26.7	Ref		
Senior		27	75.0	132	73.3	0.95	0.40, 2.27	0.904
Size of airway device^¶^								
Usual		15	41.7	62	34.4	Ref		
Large		21	58.3	118	65.6	0.52	0.10, 2.88	0.457
Duration of anesthesia (h)						1.00	0.99, 1.01	0.577

AOR: adjusted odd ratio; CI: confidence interval

^†^The types of head-and-neck surgery are described in Table 1.

^§^Clinical anesthesia experience (in years) of operator for intubation was defined as junior (<5 years) or senior (≥5 years).

^‡^Difficult intubation was defined as failure to establish a secure airway (i.e. endotracheal tube or LMA) after 3 attempts by a senior anesthetist.

^¶^Size of airway device was defined as usual (endotracheal tube ID ≤7.0mm for female and ID ≤7.5mm for male; LMA ≤3# for female and ≤4# for male) or large (any device size larger than those defined as usual in female and male).

*P< 0.05 is considered as statistically significant.

**Table 5:** Conditional logistic regression analysis of the risk factors associated with postoperative lingual neuropraxy (cases vs non-cases population, n = 36 vs 54314).

Characteristics	Univariate			Multivariate		
	OR	95% CI	P	OR	95% CI	P
Age	0.98	0.96, 0.99	0.002*			
Age group						
≦30 y/o	Ref	Ref			Ref	
30–50 y/o	0.79	0.35, 1.75	0.558	0.98	0.44, 2.20	0.964
50–70 y/o	0.35	0.14, 0.86	0.022*	0.41	0.17, 1.03	0.057
>70 y/o	0.16	0.03, 0.72	0.017*	0.33	0.07, 1.58	0.167
Gender						
Male	Ref	Ref				
Female	1.72	0.89, 3.34	0.109			
ASA PS	0.28	0.17, 0.47	<0.001*			
ASA PS class				-		-
1–2	Ref	Ref			Ref	
≥3	0.10	0.03, 0.43	0.002*	0.16	0.04, 0.68	0.014*
Site of operation^†^						
Non-head-and-neck surgery	Ref	Ref			Ref	
Head-and-neck surgery	31.13	1.89, 61.01	<0.001*	31.92	16.17, 63.02	<0.001*

ASA PS: American Society of Anesthesiologists Physical Status; CI: confidence interval; OR: odd ratio

^†^The types of head-and-neck surgery are described in Table 1; n = 53248 for non-head-and-neck surgery and n = 1102 for head-and-neck surgery.

*P< 0.05 is considered as statistically significant.

## Discussion

The present retrospective matched case-controlled study revealed a low incidence of postoperative lingual nerve injury during general anesthesia of 6.6 cases per 10,000. Risk factors found to be associated with postoperative lingual nerve injury include young age, ASA PS I-II, and head-and-neck surgery. Numerous cases of postoperative tongue numbness, or lingual neuropraxy, have been reported in anesthesia-related [2,9,12] and surgery-related [13,14] journals. As a relatively rare postoperative complication, cases of postoperative lingual neuropraxy are mostly reported in the form of case reports or case series. The exact risk factors associated with postoperative lingual neuropraxy can be difficult to isolate from case-based studies. To our knowledge, this is the first comparative study reporting the incidence and characterizing the associated risk factors for postoperative lingual neuropraxy.

Lingual nerve injury is a common, and sometimes inevitable, consequence of maxillofacial surgery, and of operations on the third molar in particular [8]. Therefore, in order to determine the independent risk factors that have not been clearly identified, we opted to exclude patients who received dental or orthognathic surgery from the analysis. The electronic postoperative registry database in Tzu Chi General Hospital only records the basic details of surgical patients (*e*.*g*. age, gender, ASA PS) and surgery-related information (*e*.*g*. types of surgery and anesthesia, duration of operation). In order to analyze potential parameters and risk factors in more detail, non-case controls from our database were selected in a 1:5 ratio after matching for age, gender, ASA PS, and anesthesia type.

The most commonly reported risk factors associated with post-anesthesia lingual nerve injury have been summarized by Thiruvenkatarajan et al. [9]. In general, more cases of postoperative lingual numbness in patients who have been anesthetized with supraglottis airway techniques (*e*.*g*. LMA) have been reported in the medical literature than patients who have received ETGA [9]. LMA-related lingual nerve injury is generally thought to result from pressure neuropraxia, with inappropriate size or misplacement of the device due to poor technique, patient positioning (lateral or prone), and cuff over-inflation of the device [2,4,9]. In this study, we tried to identify these factors from a collection of 36 patients who complained of tongue numbness after operations. The results indicated that the patients who complained of tongue numbness was made up of an equal number of patients who received ETGA or LMA (n = 18 for each), suggesting that both these commonly used airway devices may be a risk factor for postoperative lingual nerve injury. The size of the airway devices used in anesthesia maintenance did not differ among the case group in comparison to the matched controls, even after adjusting device size for patient BMI. ETGA and LMA sizes for adults (>18 years) are generally selected in accordance to patient gender, rather than body weight or other parameters in the Hualien Tzu Chi General Hospital. Our general guides for airway device selection suggest size 7.0-mm (ID) endotracheal tubes or #3 LMA for female adults, and size 7.5-mm (ID) endotracheal tubes or #4 LMA for male adults, as the recommended device sizes are typically larger for the western adults [15,16]. As a result of the routine use of smaller airway devices, a potentially higher incidence of postoperative lingual neuropraxy in patients who received general anesthesia via laryngeal masks may have been masked. Another common anesthesia-related factor is the technique and experience of anesthetists who operate the airway instrumentation. Anesthetists were classified by years of experience as either junior operators (<5 years of experience in clinical anesthesia) or senior operators (<5 years of experience in clinical anesthesia). Our analysis showed that the extent of clinical experience did not affect the occurrence of lingual neuropraxy. However, the method of quantification of clinical experience and intubation skill in this study was unconscientious, it was unanimously agreed that airway instrumentation operations should be performed by experienced personnel or under appropriate supervision to avoid unwanted cranial nerve injury [9]. It was also speculated that the duration of airway device in place did not affect the incidence of postoperative tongue numbness, as no difference in average time under anesthesia was found between the case and non-case matched control groups.

Direct invasive procedures in the molar regions, strain and traction forces resulting from surgical procedures, site of operation, and patient positioning have all been suggested as important risk factors of postoperative tongue numbness [2.9]. Patients who underwent head-and-neck surgeries were associated with a significantly higher incidence of tongue numbness when compare to patients who underwent surgeries on other sites. Multivariate logistic regression analysis showed that the adjusted odds ratio of developing tongue numbness was 7.63 (95% CI 2.03–28.70) in head-and-neck surgeries. The analysis also found that the positioning of patients during operations affected the occurrence of lingual nerve injury. There were more cases of tongue numbness in patients who were placed in supine or lateral positions. However, the difference became insignificant following multivariate logistic regression analysis, suggesting positioning may not be an independent risk factor for tongue numbness. Therefore, lingual nerve injury is most likely caused by the excessive tissue strain or traction forces generated by surgical manipulation on the head-and-neck regions which transfer extraneous mechanical pressure on the tongue tissue through the airway device (endotracheal tube or laryngeal mask) in situ.

Previous case reports have suggested that patient characteristics may contribute to the development of postoperative tongue numbness [9]. Our study found that patients who developed postoperative lingual numbness were significantly younger and healthier with lower ASA PS when compared to the entire surgical population at risk. It is reasonable that younger patients are typically associated with fewer comorbidities (i.e. lower ASA PS). To further confirm the interactions between head-and-neck surgery and the three characteristic parameters (i.e. age, gender, and ASA PS) that were matched in the second stage of analysis, a multivariate regression analysis was performed. The analysis confirmed that head-and-neck surgery and lower ASA PS are two independent risk factors for postoperative lingual neuropraxy, but the incidences of tongue numbness were not significantly different between age groups in the multivariate analysis. We believe that the standards for perioperative care are identical across all age groups in our hospital and so it was assumed that the difference observed is not a reflection of a difference in quality-of-care during operations. It was not possible to establish a direct causal relationship for the physiology of reduced incidence of lingual neuropraxy secondary to airway instrumentation in older patients with the present retrospective study design. However, atrophy of oral mucosa and soft tissues in the elderly may lead to less pressure opposing the airway device on the tongue compared to younger patients with more voluminous soft tissue in the oral cavity [17,18]. However, the causal relationships between age, ASA PS class and lingual neuropraxy require further clinical investigation.

This study was subject to a number of limitations. First, missing or lost patient records may lead to an underestimation of incidence in retrospective design studies. However, tongue numbness is inevitably associated with dysphonia that could be easily detected during postanesthesia visits. Second, our study did not compare the outcomes for different endotracheal tubes and laryngeal masks available in the market. Third, cuff pressure or volume of the laryngeal masks used for anesthesia were not routinely recorded. Therefore, the potential effect of overinflated cuffs on the incidence of lingual neuropraxy was not demonstrated. Finally, surgical patients were not routinely followed up after discharge from our hospital. Therefore, we are not able to present the recovery time and long-term consequences of postoperative tongue numbness. Nevertheless, the length of hospital stay was similar between the two groups, indicating that this postoperative event was apparently not severe enough to result in prolonged hospitalization.

Using a two-stage analysis, this retrospective study highlights several important issues in the development of postoperative lingual neuropraxy. Head-and-neck operations and patient factors (age and ASA PS) were the most identifiable risk factors for this unwanted postoperative event. Although our analysis did not detect any significant impact of anesthesia-related factors on this adverse event, it remains plausible that pressure opposed by an airway device on the tongue may contribute to lingual neuropraxy. Therefore, we emphasize that the prevention of postoperative lingual neuropraxy should focus on the identification of associating risk factors, particularly in reducing pressure of endotracheal tubes and laryngeal masks on the tongue during head-and-neck operations to avert the occurrence of postoperative lingual neuropraxy, provided that the optimal but smallest possible size of airway device is chosen.

## Supporting information

S1 Table Clinical information of patients with post-operative lingual neuropraxy. (DOCX)

S2 Table Minimal dataset for statistical analysis. (PDF)

S3 Table Codebook for minimal dataset. (PDF)
