# Supplementary material for: Incidence and risk factors for postoperative lingual neuropraxia following airway instrumentation: A retrospective matched case-control study
Source: PLoS One. 2018 Jan 12;13(1):e0190589. doi: 10.1371/journal.pone.0190589 (PMC5766107; doi:10.1371/journal.pone.0190589)
Supplement: S1 Table — (DOCX) [file pone.0190589.s001.docx]

**Supplementary Table 1.**

| ID | Age | Gender | ASA PS | Airway device | Size of device | Anesthesia time (min) | Operator* | Position | Operation | taste disturbance |
| --- | --- | --- | --- | --- | --- | --- | --- | --- | --- | --- |
| 1 | 19 | M | 1 | LMA | 4 | 70 | senior | supine | Patella open fracture for ORIF | no |
| 2 | 72 | F | 3 | ETGA | 7 | 175 | junior | supine | Parathyroidectomy | yes |
| 3 | 42 | M | 2 | ETGA | 7 | 300 | senior | supine | Cervical 3-6 laminectomy | no |
| 4 | 36 | M | 1 | LMA | 4 | 50 | senior | supine | Removal of internal fixator in ankle | no |
| 5 | 58 | M | 2 | ETGA | 7.5 | 70 | senior | supine | Submandibular sialolithiasis excision | no |
| 6 | 60 | F | 2 | ETGA | 7 | 215 | senior | supine | Tympanoplasty | no |
| 7 | 26 | F | 1 | ETGA | 7 | 45 | junior | supine | Apocrinectomy | no |
| 8 | 27 | F | 1 | LMA | 3 | 85 | senior | supine | Debridement | no |
| 9 | 46 | M | 3 | ETGA | 7.5 | 185 | senior | lateral | Revision total hip replacement | no |
| 10 | 30 | M | 2 | ETGA | 7.5 | 125 | junior | supine | Uvulopalatopharyngoplasty | no |
| 11 | 61 | F | 2 | ETGA | 7.0 | 85 | senior | supine | Tonsillectomy | no |
| 12 | 33 | M | 1 | ETGA | 7.5 | 110 | senior | supine | Functional endoscopic sinus surgery | no |
| 13 | 52 | F | 1 | ETGA | 5 | 75 | senior | supine | Tosillectomy | no |
| 14 | 46 | F | 2 | LMA | 3 | 35 | senior | lithotomy | Cystoscopic surgery | no |
| 15 | 39 | M | 2 | LMA | 4 | 185 | junior | lateral | Removal of internal fixator in femur | no |
| 16 | 29 | F | 2 | LMA | 3 | 95 | senior | supine | Removal of internal fixator in humerus | no |
| 17 | 55 | F | 2 | ETGA | 7 | 155 | senior | supine | Thyroidectomy | no |
| 18 | 28 | M | 1 | ETGA | 7 | 155 | senior | supine | Tonsillectomy | no |
| 19 | 55 | F | 1 | ETGA | 7 | 180 | senior | supine | Laparoscopic Assisted Vaginal Hysterectomy | no |
| 20 | 40 | M | 1 | LMA | 4 | 135 | junior | supine | Ankle fusion | no |
| 21 | 38 | F | 2 | ETGA | 7 | 265 | NA | supine | Thyroidectomy | no |
| 22 | 48 | M | 2 | LMA | 4 | 75 | NA | supine | Debridement | no |
| 23 | 28 | F | 1 | LMA | 3 | 90 | junior | lateral | Removal of internal fixator in femur | no |
| 24 | 24 | F | 1 | LMA | 3 | 70 | NA | supine | Debridement | no |
| 25 | 47 | F | 1 | LMA | 3 | 95 | NA | supine | Lateral malleollar fracture for ORIF | no |
| 26 | 72 | M | 2 | ETGA | 7.5 | 150 | NA | supine | Parotic tumor excision | no |
| 27 | 57 | F | 1 | LMA | 3 | 70 | NA | supine | Laparoscopic appendectomy | no |
| 28 | 34 | M | 2 | ETGA | 7.5 | 115 | NA | supine | Zygomatic fracture for ORIF | no |
| 29 | 58 | F | 2 | ETGA | 7 | 205 | NA | supine | Tympanoplasty | no |
| 30 | 36 | F | 2 | LMA | 3 | 220 | junior | supine | Debridement + external skeletal fixation | no |
| 31 | 19 | F | 1 | LMA | 3 | 95 | NA | lateral | Elbow fracture for ORIF | no |
| 32 | 56 | F | 2 | ETGA | 7 | 220 | junior | supine | Breast tumor excision | no |
| 33 | 47 | F | 2 | LMA | 3 | 100 | NA | lateral | Debridement | no |
| 34 | 38 | F | 2 | LMA | 3 | 215 | NA | supine | Forearm wound repair | no |
| 35 | 19 | M | 1 | LMA | 4 | 185 | NA | supine | Knee arthroscope | no |
| 36 | 42 | M | 1 | LMA | 3 | 85 | junior | supine | Herniorrhaphy | no |

ASA PS: American Society of Anesthesiologists physical status; ET: endotracheal tube; Lateral: lateral decubitus; LM: laryngeal mask; NA: not available; ORIF: open reduction and internal fixation. *clinical anesthesia experience of operator was defined as junior (< 5 years of experience) or senior (≥ 5 years of experience).
