# Supplementary material for: Incidence and risk factors for postoperative lingual neuropraxia following airway instrumentation: A retrospective matched case-control study
Source: PLoS One. 2018 Jan 12;13(1):e0190589. doi: 10.1371/journal.pone.0190589 (PMC5766107; doi:10.1371/journal.pone.0190589)
Supplement: S2 Table — (PDF) [file pone.0190589.s002.pdf]

| Group | No | Gender | Age | Age_Group | Height | Weight | BMI | BMI_Group | OP_site | ASA |
|---|---|---|---|---|---|---|---|---|---|---|
| 1 | 1 | 1 | 19 | 1 | 155 | 47 | 19.6 | 1 | 0 | 1 |
| 1 | 2 | 2 | 72 | 4 | 145 | 52 | 24.7 | 3 | 1 | 3 |
| 1 | 3 | 1 | 42 | 2 | 164 | 93.6 | 34.8 | 3 | 1 | 2 |
| 1 | 4 | 1 | 36 | 2 | 183 | 74 | 22.1 | 1 | 0 | 1 |
| 1 | 5 | 1 | 58 | 3 | 171 | 74 | 25.3 | 3 | 1 | 2 |
| 1 | 6 | 2 | 60 | 3 | 153.5 | 58.1 | 24.7 | 3 | 1 | 2 |
| 1 | 7 | 2 | 26 | 1 | 163 | 54 | 20.3 | 1 | 0 | 1 |
| 1 | 8 | 2 | 27 | 1 | 159 | 52.4 | 20.7 | 1 | 0 | 1 |
| 1 | 9 | 1 | 46 | 2 | 166 | 140 | 50.8 | 3 | 0 | 3 |
| 1 | 10 | 1 | 30 | 1 | 177.5 | 95.5 | 30.3 | 3 | 1 | 2 |
| 1 | 11 | 2 | 61 | 3 | 149 | 68.9 | 31.0 | 3 | 1 | 2 |
| 1 | 12 | 1 | 33 | 2 | 175 | 83 | 27.1 | 3 | 1 | 1 |
| 1 | 13 | 2 | 52 | 3 | 153 | 58.4 | 24.9 | 3 | 1 | 1 |
| 1 | 14 | 2 | 46 | 2 | 159 | 56 | 22.1 | 1 | 0 | 2 |
| 1 | 15 | 1 | 39 | 2 | 175 | 65 | 21.2 | 1 | 0 | 2 |
| 1 | 16 | 2 | 29 | 1 | 166 | 48.9 | 17.7 | 2 | 0 | 2 |
| 1 | 17 | 2 | 55 | 3 | 160 | 83.5 | 32.6 | 3 | 1 | 2 |
| 1 | 18 | 1 | 28 | 1 | 175.3 | 74.6 | 24.3 | 3 | 1 | 1 |
| 1 | 19 | 2 | 55 | 3 | 168.3 | 58.55 | 20.7 | 1 | 0 | 1 |
| 1 | 20 | 1 | 40 | 2 | 170.5 | 73.25 | 25.2 | 3 | 0 | 1 |
| 1 | 21 | 2 | 38 | 2 | 155.5 | 56 | 23.2 | 1 | 1 | 2 |
| 1 | 22 | 1 | 48 | 2 | 170 | 83 | 28.7 | 3 | 0 | 2 |
| 1 | 23 | 2 | 28 | 1 | 158 | 56 | 22.4 | 1 | 0 | 1 |
| 1 | 24 | 2 | 24 | 1 | 171 | 56.7 | 19.4 | 1 | 0 | 1 |
| 1 | 25 | 2 | 47 | 2 | 152.5 | 61 | 26.2 | 3 | 0 | 1 |
| 1 | 26 | 1 | 71 | 4 | 160 | 43.75 | 17.1 | 2 | 1 | 2 |
| 1 | 27 | 2 | 57 | 3 | 161.5 | 73.5 | 28.2 | 3 | 0 | 1 |
| 1 | 28 | 1 | 34 | 2 | 174 | 106.7 | 35.2 | 3 | 1 | 2 |
| 1 | 29 | 2 | 58 | 3 | 150 | 72.4 | 32.2 | 3 | 1 | 2 |
| 1 | 30 | 2 | 36 | 2 | 160 | 75 | 29.3 | 3 | 0 | 2 |
| 1 | 31 | 2 | 19 | 1 | 160 | 46 | 18.0 | 2 | 0 | 1 |
| 1 | 32 | 2 | 56 | 3 | 150.4 | 54 | 23.9 | 1 | 0 | 2 |
| 1 | 33 | 2 | 47 | 2 | 156 | 60 | 24.7 | 3 | 0 | 2 |
| 1 | 34 | 2 | 38 | 2 | 162 | 80 | 30.5 | 3 | 0 | 2 |
| 1 | 35 | 1 | 19 | 1 | 178 | 80 | 25.2 | 3 | 0 | 1 |
| 1 | 36 | 1 | 42 | 2 | 162 | 63.6 | 24.2 | 3 | 0 | 1 |
| 0 | 1 | 1 | 19 | 1 | 156 | 75 | 30.8 | 3 | 1 | 1 |
| 0 | 1 | 1 | 19 | 1 | 175 | 70 | 22.9 | 1 | 0 | 1 |
| 0 | 1 | 1 | 19 | 1 | 170 | 66.6 | 23.0 | 1 | 0 | 1 |
| 0 | 1 | 1 | 19 | 1 | 173 | 66 | 22.1 | 1 | 0 | 1 |
| 0 | 1 | 1 | 19 | 1 | 174.5 | 106.9 | 35.1 | 3 | 0 | 1 |
| 0 | 2 | 2 | 72 | 4 | 143 | 55.9 | 27.3 | 3 | 0 | 3 |
| 0 | 2 | 2 | 72 | 4 | 146 | 51 | 23.9 | 1 | 1 | 3 |
| 0 | 2 | 2 | 72 | 4 | 151.5 | 47 | 20.5 | 1 | 1 | 3 |
| 0 | 2 | 2 | 72 | 4 | 153 | 54 | 23.1 | 1 | 1 | 3 |
| 0 | 2 | 2 | 72 | 4 | 148 | 41.7 | 19.0 | 1 | 0 | 3 |
| 0 | 3 | 1 | 42 | 2 | 168 | 82 | 29.1 | 3 | 1 | 2 |
| 0 | 3 | 1 | 42 | 2 | 169.5 | 78 | 27.1 | 3 | 1 | 2 |
| 0 | 3 | 1 | 42 | 2 | 174.1 | 86.7 | 28.6 | 3 | 1 | 2 |
| 0 | 3 | 1 | 42 | 2 | 170 | 65 | 22.5 | 1 | 1 | 2 |
| 0 | 3 | 1 | 42 | 2 | 164 | 85 | 31.6 | 3 | 0 | 2 |
| 0 | 4 | 1 | 36 | 2 | 170 | 76 | 26.3 | 3 | 0 | 1 |
| 0 | 4 | 1 | 36 | 2 | 174 | 75.2 | 24.8 | 3 | 0 | 1 |
| 0 | 4 | 1 | 36 | 2 | 173.8 | 87.9 | 29.1 | 3 | 0 | 1 |
| 0 | 4 | 1 | 36 | 2 | 189.5 | 68.35 | 19.0 | 1 | 0 | 1 |
| 0 | 4 | 1 | 36 | 2 | 165 | 82 | 30.1 | 3 | 0 | 1 |

| | | | | | | | | | | |
|---|---|---|---|---|---|---|---|---|---|---|
| 0 | 5 | 1 | 58 | 3 | 164 | 63.6 | 23.6 | 1 | 0 | 2 |
| 0 | 5 | 1 | 58 | 3 | 167.5 | 66 | 23.5 | 1 | 1 | 2 |
| 0 | 5 | 1 | 58 | 3 | 160 | 58 | 22.7 | 1 | 0 | 2 |
| 0 | 5 | 1 | 58 | 3 | 161.8 | 83.2 | 31.8 | 3 | 0 | 2 |
| 0 | 5 | 1 | 58 | 3 | 159 | 62 | 24.5 | 3 | 0 | 2 |
| 0 | 6 | 2 | 60 | 3 | 153.5 | 48 | 20.4 | 1 | 0 | 2 |
| 0 | 6 | 2 | 60 | 3 | 161 | 66.5 | 25.7 | 3 | 0 | 2 |
| 0 | 6 | 2 | 60 | 3 | 158 | 66 | 26.4 | 3 | 1 | 2 |
| 0 | 6 | 2 | 60 | 3 | 157.8 | 60.3 | 24.2 | 3 | 0 | 2 |
| 0 | 6 | 2 | 60 | 3 | 155 | 55 | 22.9 | 1 | 0 | 2 |
| 0 | 7 | 2 | 26 | 1 | 168 | 70 | 24.8 | 3 | 0 | 1 |
| 0 | 7 | 2 | 26 | 1 | 160.8 | 57.5 | 22.2 | 1 | 0 | 1 |
| 0 | 7 | 2 | 26 | 1 | 150 | 45.4 | 20.2 | 1 | 0 | 1 |
| 0 | 7 | 2 | 26 | 1 | 169.5 | 58.7 | 20.4 | 1 | 0 | 1 |
| 0 | 7 | 2 | 26 | 1 | 152 | 45 | 19.5 | 1 | 0 | 1 |
| 0 | 8 | 2 | 27 | 1 | 152 | 47.45 | 20.5 | 1 | 0 | 1 |
| 0 | 8 | 2 | 27 | 1 | 150 | 64 | 28.4 | 3 | 0 | 1 |
| 0 | 8 | 2 | 27 | 1 | 164 | 58 | 21.6 | 1 | 0 | 1 |
| 0 | 8 | 2 | 27 | 1 | 162.5 | 61.6 | 23.3 | 1 | 0 | 1 |
| 0 | 8 | 2 | 27 | 1 | 153.5 | 54.2 | 23.0 | 1 | 0 | 1 |
| 0 | 9 | 1 | 46 | 2 | 156 | 79.4 | 32.6 | 3 | 0 | 3 |
| 0 | 9 | 1 | 46 | 2 | 170 | 59.5 | 20.6 | 1 | 0 | 3 |
| 0 | 9 | 1 | 46 | 2 | 180 | 66 | 20.4 | 1 | 1 | 3 |
| 0 | 9 | 1 | 46 | 2 | 179 | 61.5 | 19.2 | 1 | 1 | 3 |
| 0 | 9 | 1 | 46 | 2 | 170 | 80 | 27.7 | 3 | 1 | 3 |
| 0 | 10 | 1 | 30 | 1 | 163 | 75 | 28.2 | 3 | 0 | 2 |
| 0 | 10 | 1 | 30 | 1 | 170 | 75 | 26.0 | 3 | 0 | 2 |
| 0 | 10 | 1 | 30 | 1 | 164 | 57 | 21.2 | 1 | 1 | 2 |
| 0 | 10 | 1 | 30 | 1 | 182 | 80 | 24.2 | 3 | 0 | 2 |
| 0 | 10 | 1 | 30 | 1 | 179 | 108.2 | 33.8 | 3 | 0 | 2 |
| 0 | 11 | 2 | 61 | 3 | 162.5 | 72.1 | 27.3 | 3 | 0 | 2 |
| 0 | 11 | 2 | 61 | 3 | 149 | 36 | 16.2 | 2 | 0 | 2 |
| 0 | 11 | 2 | 61 | 3 | 155 | 75 | 31.2 | 3 | 0 | 2 |
| 0 | 11 | 2 | 61 | 3 | 161 | 84 | 32.4 | 3 | 0 | 2 |
| 0 | 11 | 2 | 61 | 3 | 157 | 55.6 | 22.6 | 1 | 0 | 2 |
| 0 | 12 | 1 | 33 | 2 | 176 | 77 | 24.9 | 3 | 1 | 1 |
| 0 | 12 | 1 | 33 | 2 | 170 | 72.15 | 25.0 | 3 | 0 | 1 |
| 0 | 12 | 1 | 33 | 2 | 170.4 | 88.7 | 30.5 | 3 | 1 | 1 |
| 0 | 12 | 1 | 33 | 2 | 163 | 82 | 30.9 | 3 | 0 | 1 |
| 0 | 12 | 1 | 33 | 2 | 174 | 70 | 23.1 | 1 | 0 | 1 |
| 0 | 13 | 2 | 52 | 3 | 155.5 | 66 | 27.3 | 3 | 1 | 1 |
| 0 | 13 | 2 | 52 | 3 | 167 | 64 | 22.9 | 1 | 0 | 1 |
| 0 | 13 | 2 | 52 | 3 | 154 | 55 | 23.2 | 1 | 0 | 1 |
| 0 | 13 | 2 | 52 | 3 | 159 | 60 | 23.7 | 1 | 0 | 1 |
| 0 | 13 | 2 | 52 | 3 | 157.5 | 55 | 22.2 | 1 | 0 | 1 |
| 0 | 14 | 2 | 46 | 2 | 151.6 | 63.6 | 27.7 | 3 | 0 | 2 |
| 0 | 14 | 2 | 46 | 2 | 155.6 | 61.7 | 25.5 | 3 | 0 | 2 |
| 0 | 14 | 2 | 46 | 2 | 156.5 | 55.9 | 22.8 | 1 | 0 | 2 |
| 0 | 14 | 2 | 46 | 2 | 155 | 76 | 31.6 | 3 | 0 | 2 |
| 0 | 14 | 2 | 46 | 2 | 148 | 45 | 20.5 | 1 | 0 | 2 |
| 0 | 15 | 1 | 39 | 2 | 172 | 72 | 24.3 | 3 | 0 | 2 |
| 0 | 15 | 1 | 39 | 2 | 166.5 | 70 | 25.3 | 3 | 0 | 2 |
| 0 | 15 | 1 | 39 | 2 | 168 | 70 | 24.8 | 3 | 0 | 2 |
| 0 | 15 | 1 | 39 | 2 | 162 | 52 | 19.8 | 1 | 0 | 2 |
| 0 | 15 | 1 | 39 | 2 | 172 | 70 | 23.7 | 1 | 0 | 2 |
| 0 | 16 | 2 | 29 | 1 | 164 | 61.5 | 22.9 | 1 | 0 | 2 |
| 0 | 16 | 2 | 29 | 1 | 158 | 55 | 22.0 | 1 | 0 | 2 |

| | | | | | | | | | | |
|---|---|---|---|---|---|---|---|---|---|---|
| 0 | 16 | 2 | 29 | 1 | 159 | 63 | 24.9 | 3 | 0 | 2 |
| 0 | 16 | 2 | 29 | 1 | 153 | 44.2 | 18.9 | 1 | 0 | 2 |
| 0 | 16 | 2 | 29 | 1 | 155.8 | 42.8 | 17.6 | 2 | 0 | 2 |
| 0 | 17 | 2 | 55 | 3 | 154 | 55.6 | 23.4 | 1 | 0 | 2 |
| 0 | 17 | 2 | 55 | 3 | 160 | 65 | 25.4 | 3 | 0 | 2 |
| 0 | 17 | 2 | 55 | 3 | 162 | 50 | 19.1 | 1 | 0 | 2 |
| 0 | 17 | 2 | 55 | 3 | 156 | 63 | 25.9 | 3 | 1 | 2 |
| 0 | 17 | 2 | 55 | 3 | 165 | 65 | 23.9 | 1 | 0 | 2 |
| 0 | 18 | 1 | 28 | 1 | 163.2 | 52.5 | 19.7 | 1 | 1 | 1 |
| 0 | 18 | 1 | 28 | 1 | 178 | 88 | 27.8 | 3 | 0 | 1 |
| 0 | 18 | 1 | 28 | 1 | 175 | 81.3 | 26.5 | 3 | 0 | 1 |
| 0 | 18 | 1 | 28 | 1 | 172.5 | 55 | 18.5 | 2 | 0 | 1 |
| 0 | 18 | 1 | 28 | 1 | 174 | 84 | 27.7 | 3 | 0 | 1 |
| 0 | 19 | 2 | 55 | 3 | 160 | 59.1 | 23.1 | 1 | 1 | 1 |
| 0 | 19 | 2 | 55 | 3 | 165 | 54 | 19.8 | 1 | 1 | 1 |
| 0 | 19 | 2 | 55 | 3 | 155 | 64.4 | 26.8 | 3 | 0 | 1 |
| 0 | 19 | 2 | 55 | 3 | 161 | 67 | 25.8 | 3 | 0 | 1 |
| 0 | 19 | 2 | 55 | 3 | 151 | 57 | 25.0 | 3 | 0 | 1 |
| 0 | 20 | 1 | 40 | 2 | 160.5 | 54 | 21.0 | 1 | 0 | 1 |
| 0 | 20 | 1 | 40 | 2 | 166.2 | 85.9 | 31.1 | 3 | 0 | 1 |
| 0 | 20 | 1 | 40 | 2 | 173 | 75.2 | 25.1 | 3 | 0 | 1 |
| 0 | 20 | 1 | 40 | 2 | 177.3 | 83.7 | 26.6 | 3 | 0 | 1 |
| 0 | 20 | 1 | 40 | 2 | 168 | 70 | 24.8 | 3 | 0 | 1 |
| 0 | 21 | 2 | 38 | 2 | 153 | 85.9 | 36.7 | 3 | 0 | 2 |
| 0 | 21 | 2 | 38 | 2 | 149 | 53.6 | 24.1 | 3 | 0 | 2 |
| 0 | 21 | 2 | 38 | 2 | 162.8 | 49.7 | 18.8 | 1 | 1 | 2 |
| 0 | 21 | 2 | 38 | 2 | 162 | 82.5 | 31.4 | 3 | 1 | 2 |
| 0 | 21 | 2 | 38 | 2 | 155.2 | 64.1 | 26.6 | 3 | 0 | 2 |
| 0 | 22 | 1 | 48 | 2 | 166 | 63 | 22.9 | 1 | 0 | 2 |
| 0 | 22 | 1 | 48 | 2 | 166.5 | 66.65 | 24.0 | 3 | 0 | 2 |
| 0 | 22 | 1 | 48 | 2 | 167 | 63.8 | 22.9 | 1 | 0 | 2 |
| 0 | 22 | 1 | 48 | 2 | 164 | 58 | 21.6 | 1 | 0 | 2 |
| 0 | 22 | 1 | 48 | 2 | 172 | 74 | 25.0 | 3 | 0 | 2 |
| 0 | 23 | 2 | 28 | 1 | 162.5 | 55.1 | 20.9 | 1 | 0 | 1 |
| 0 | 23 | 2 | 28 | 1 | 157 | 107 | 43.4 | 3 | 0 | 1 |
| 0 | 23 | 2 | 28 | 1 | 161 | 46 | 17.7 | 2 | 0 | 1 |
| 0 | 23 | 2 | 28 | 1 | 155 | 51 | 21.2 | 1 | 0 | 1 |
| 0 | 23 | 2 | 28 | 1 | 150 | 47 | 20.9 | 1 | 0 | 1 |
| 0 | 24 | 2 | 24 | 1 | 168 | 58 | 20.5 | 1 | 0 | 1 |
| 0 | 24 | 2 | 24 | 1 | 156 | 60 | 24.7 | 3 | 0 | 1 |
| 0 | 24 | 2 | 24 | 1 | 159 | 49 | 19.4 | 1 | 0 | 1 |
| 0 | 24 | 2 | 24 | 1 | 150.2 | 64.4 | 28.5 | 3 | 0 | 1 |
| 0 | 24 | 2 | 24 | 1 | 157 | 60 | 24.3 | 3 | 0 | 1 |
| 0 | 25 | 2 | 47 | 2 | 167 | 57.3 | 20.5 | 1 | 0 | 1 |
| 0 | 25 | 2 | 47 | 2 | 149.2 | 56.8 | 25.5 | 3 | 0 | 1 |
| 0 | 25 | 2 | 47 | 2 | 158 | 77 | 30.8 | 3 | 0 | 1 |
| 0 | 25 | 2 | 47 | 2 | 164 | 70 | 26.0 | 3 | 0 | 1 |
| 0 | 25 | 2 | 47 | 2 | 148 | 73 | 33.3 | 3 | 0 | 1 |
| 0 | 26 | 1 | 71 | 4 | 169.7 | 74.2 | 25.8 | 3 | 1 | 2 |
| 0 | 26 | 1 | 71 | 4 | 160 | 64.5 | 25.2 | 3 | 0 | 2 |
| 0 | 26 | 1 | 71 | 4 | 158 | 58.3 | 23.4 | 1 | 0 | 2 |
| 0 | 26 | 1 | 71 | 4 | 168.7 | 59 | 20.7 | 1 | 0 | 2 |
| 0 | 26 | 1 | 71 | 4 | 168 | 71 | 25.2 | 3 | 0 | 2 |
| 0 | 27 | 2 | 57 | 3 | 147.5 | 51.5 | 23.7 | 1 | 0 | 1 |
| 0 | 27 | 2 | 57 | 3 | 155 | 66 | 27.5 | 3 | 0 | 1 |
| 0 | 27 | 2 | 57 | 3 | 156 | 72.3 | 29.7 | 3 | 0 | 1 |
| 0 | 27 | 2 | 57 | 3 | 156 | 62 | 25.5 | 3 | 0 | 1 |

| | | | | | | | | | | |
|---|---|---|---|---|---|---|---|---|---|---|
| 0 | 27 | 2 | 57 | 3 | 168.5 | 78.5 | 27.6 | 3 | 0 | 1 |
| 0 | 28 | 1 | 34 | 2 | 164 | 66 | 24.5 | 3 | 1 | 2 |
| 0 | 28 | 1 | 34 | 2 | 180 | 48 | 14.8 | 2 | 0 | 2 |
| 0 | 28 | 1 | 34 | 2 | 180.7 | 70.6 | 21.6 | 1 | 1 | 2 |
| 0 | 28 | 1 | 34 | 2 | 178.3 | 93 | 29.3 | 3 | 0 | 2 |
| 0 | 28 | 1 | 34 | 2 | 165 | 69 | 25.3 | 3 | 1 | 2 |
| 0 | 29 | 2 | 58 | 3 | 151 | 55 | 24.1 | 3 | 1 | 2 |
| 0 | 29 | 2 | 58 | 3 | 151 | 54.6 | 23.9 | 1 | 0 | 2 |
| 0 | 29 | 2 | 58 | 3 | 155 | 47 | 19.6 | 1 | 0 | 2 |
| 0 | 29 | 2 | 58 | 3 | 154 | 63.9 | 26.9 | 3 | 0 | 2 |
| 0 | 29 | 2 | 58 | 3 | 166 | 59 | 21.4 | 1 | 0 | 2 |
| 0 | 30 | 2 | 36 | 2 | 157.5 | 78 | 31.4 | 3 | 0 | 2 |
| 0 | 30 | 2 | 36 | 2 | 161 | 92 | 35.5 | 3 | 0 | 2 |
| 0 | 30 | 2 | 36 | 2 | 158.4 | 51.9 | 20.7 | 1 | 0 | 2 |
| 0 | 30 | 2 | 36 | 2 | 160.7 | 56.9 | 22.0 | 1 | 0 | 2 |
| 0 | 30 | 2 | 36 | 2 | 159 | 44.6 | 17.6 | 2 | 0 | 2 |
| 0 | 31 | 2 | 19 | 1 | 166 | 47 | 17.1 | 2 | 0 | 1 |
| 0 | 31 | 2 | 19 | 1 | 159 | 43 | 17.0 | 2 | 0 | 1 |
| 0 | 31 | 2 | 19 | 1 | 154 | 76 | 32.0 | 3 | 0 | 1 |
| 0 | 31 | 2 | 19 | 1 | 158 | 60 | 24.0 | 3 | 0 | 1 |
| 0 | 31 | 2 | 19 | 1 | 162.5 | 49.9 | 18.9 | 1 | 0 | 1 |
| 0 | 32 | 2 | 56 | 3 | 156.5 | 42.9 | 17.5 | 2 | 0 | 2 |
| 0 | 32 | 2 | 56 | 3 | 156 | 63.8 | 26.2 | 3 | 0 | 2 |
| 0 | 32 | 2 | 56 | 3 | 154.6 | 66.9 | 28.0 | 3 | 0 | 2 |
| 0 | 32 | 2 | 56 | 3 | 155.2 | 72 | 29.9 | 3 | 0 | 2 |
| 0 | 32 | 2 | 56 | 3 | 153 | 62 | 26.5 | 3 | 0 | 2 |
| 0 | 33 | 2 | 47 | 2 | 156.5 | 48 | 19.6 | 1 | 0 | 2 |
| 0 | 33 | 2 | 47 | 2 | 156.5 | 50.3 | 20.5 | 1 | 0 | 2 |
| 0 | 33 | 2 | 47 | 2 | 155 | 56 | 23.3 | 1 | 0 | 2 |
| 0 | 33 | 2 | 47 | 2 | 160 | 56.2 | 22.0 | 1 | 0 | 2 |
| 0 | 33 | 2 | 47 | 2 | 155.2 | 66.4 | 27.6 | 3 | 0 | 2 |
| 0 | 34 | 2 | 38 | 2 | 159 | 72.3 | 28.6 | 3 | 0 | 2 |
| 0 | 34 | 2 | 38 | 2 | 164.5 | 54 | 20.0 | 1 | 0 | 2 |
| 0 | 34 | 2 | 38 | 2 | 162 | 77 | 29.3 | 3 | 0 | 2 |
| 0 | 34 | 2 | 38 | 2 | 166 | 53.2 | 19.3 | 1 | 0 | 2 |
| 0 | 34 | 2 | 38 | 2 | 150 | 35 | 15.6 | 2 | 0 | 2 |
| 0 | 35 | 1 | 19 | 1 | 166 | 80 | 29.0 | 3 | 0 | 1 |
| 0 | 35 | 1 | 19 | 1 | 168 | 63 | 22.3 | 1 | 0 | 1 |
| 0 | 35 | 1 | 19 | 1 | 170 | 89 | 30.8 | 3 | 0 | 1 |
| 0 | 35 | 1 | 19 | 1 | 178 | 93 | 29.4 | 3 | 0 | 1 |
| 0 | 35 | 1 | 19 | 1 | 170.8 | 78.2 | 26.8 | 3 | 0 | 1 |
| 0 | 36 | 1 | 42 | 2 | 170.6 | 95.2 | 32.7 | 3 | 0 | 1 |
| 0 | 36 | 1 | 42 | 2 | 171.5 | 72.4 | 24.6 | 3 | 0 | 1 |
| 0 | 36 | 1 | 42 | 2 | 176 | 88.8 | 28.7 | 3 | 0 | 1 |
| 0 | 36 | 1 | 42 | 2 | 171 | 81.5 | 27.9 | 3 | 0 | 1 |
| 0 | 36 | 1 | 42 | 2 | 175 | 76.7 | 25.0 | 3 | 0 | 1 |

| ASA_Type | ASA_time | Size_Group | Difficulty | Experience | Posture | Fluid | LOS |
|---|---|---|---|---|---|---|---|
| 2 | 70 | 2 | 0 | 2 | 1 | 600 | 4 |
| 1 | 175 | 2 | 0 | 1 | 1 | 1800 | 12 |
| 1 | 300 | 1 | 0 | 2 | 1 | 2800 | 51 |
| 2 | 50 | 2 | 0 | 2 | 1 | 350 | 3 |
| 1 | 70 | 2 | 0 | 2 | 1 | 605 | 3 |
| 1 | 215 | 2 | 0 | 2 | 1 | 1600 | 4 |
| 1 | 45 | 2 | 0 | 1 | 1 | 300 | 1 |
| 2 | 85 | 1 | 0 | 2 | 1 | 850 | 34 |
| 1 | 185 | 2 | 1 | 2 | 2 | 3200 | 5 |
| 1 | 125 | 2 | 0 | 1 | 1 | 500 | 3 |
| 1 | 85 | 2 | 0 | 2 | 1 | 600 | 3 |
| 1 | 110 | 2 | 0 | 2 | 1 | 800 | 4 |
| 1 | 75 | 1 | 0 | 2 | 1 | 400 | 2 |
| 2 | 35 | 1 | 0 | 2 | 3 | 250 | 2 |
| 2 | 185 | 2 | 0 | 1 | 2 | 900 | 4 |
| 2 | 95 | 1 | 0 | 2 | 1 | 400 | 4 |
| 1 | 155 | 2 | 0 | 2 | 1 | 750 | 4 |
| 1 | 155 | 1 | 0 | 2 | 1 | 400 | 3 |
| 1 | 180 | 2 | 0 | 2 | 1 | 2010 | 4 |
| 2 | 135 | 2 | 0 | 1 | 1 | 600 | 4 |
| 1 | 265 | 2 | 0 | 2 | 1 | 1700 | 3 |
| 2 | 75 | 2 | 0 | 2 | 1 | 500 | 9 |
| 2 | 90 | 1 | 0 | 1 | 2 | 600 | 2 |
| 2 | 70 | 1 | 0 | 2 | 1 | 350 | 16 |
| 2 | 95 | 1 | 0 | 2 | 1 | 700 | 3 |
| 1 | 150 | 2 | 0 | 2 | 1 | 600 | 4 |
| 2 | 70 | 1 | 0 | 2 | 1 | 200 | 4 |
| 1 | 115 | 2 | 0 | 2 | 1 | 800 | 3 |
| 1 | 205 | 2 | 0 | 2 | 1 | 450 | 4 |
| 2 | 220 | 1 | 0 | 1 | 1 | 1500 | 19 |
| 2 | 95 | 1 | 0 | 2 | 2 | 350 | 7 |
| 1 | 220 | 2 | 0 | 1 | 1 | 1700 | 4 |
| 2 | 100 | 1 | 0 | 2 | 2 | 150 | 6 |
| 2 | 215 | 1 | 0 | 2 | 1 | 1000 | 6 |
| 2 | 185 | 2 | 0 | 2 | 1 | 950 | 6 |
| 2 | 85 | 1 | 1 | 1 | 1 | 350 | 4 |
| 2 | 45 | 2 | 0 | 2 | 1 | 150 | 1 |
| 2 | 105 | 2 | 0 | 2 | 1 | 450 | 66 |
| 2 | 140 | 2 | 0 | 2 | 1 | 300 | 23 |
| 2 | 155 | 2 | 0 | 2 | 1 | 1050 | 5 |
| 2 | 40 | 2 | 0 | 2 | 1 | 350 | 1 |
| 1 | 315 | 2 | 0 | 2 | 4 | 3000 | 9 |
| 1 | 465 | 2 | 0 | 2 | 1 | 3090 | 24 |
| 1 | 235 | 2 | 0 | 2 | 4 | 2430 | 8 |
| 1 | 210 | 2 | 0 | 2 | 1 | 1900 | 3 |
| 1 | 145 | 2 | 0 | 1 | 1 | 750 | 17 |
| 1 | 65 | 1 | 0 | 2 | 1 | 550 | 54 |
| 1 | 120 | 2 | 0 | 2 | 1 | 400 | 10 |
| 1 | 65 | 1 | 0 | 2 | 1 | 450 | 12 |
| 1 | 105 | 2 | 0 | 2 | 1 | 500 | 3 |
| 1 | 560 | 2 | 0 | 2 | 4 | 2350 | 21 |
| 2 | 60 | 2 | 0 | 2 | 2 | 500 | 3 |
| 2 | 95 | 2 | 0 | 2 | 1 | 300 | 1 |
| 2 | 150 | 2 | 0 | 2 | 1 | 570 | 2 |
| 2 | 80 | 2 | 0 | 1 | 1 | 350 | 4 |
| 2 | 35 | 2 | 0 | 2 | 1 | 200 | 4 |

| | | | | | | | |
|---|---|---|---|---|---|---|---|
| 1 | 430 | 2 | 0 | 2 | 1 | 1370 | 12 |
| 1 | 235 | 2 | 0 | 1 | 1 | 850 | 7 |
| 1 | 85 | 1 | 0 | 2 | 1 | 750 | 6 |
| 1 | 60 | 2 | 0 | 2 | 4 | 600 | 4 |
| 1 | 115 | 2 | 0 | 2 | 1 | 350 | 6 |
| 1 | 180 | 2 | 0 | 2 | 1 | 1680 | 7 |
| 1 | 160 | 2 | 0 | 2 | 1 | 1900 | 4 |
| 1 | 150 | 2 | 0 | 1 | 1 | 1100 | 3 |
| 1 | 120 | 2 | 0 | 1 | 4 | 1000 | 7 |
| 1 | 312 | 2 | 0 | 2 | 4 | 5000 | 7 |
| 1 | 95 | 2 | 0 | 2 | 1 | 800 | 3 |
| 1 | 70 | 2 | 0 | 2 | 1 | 350 | 3 |
| 1 | 220 | 2 | 0 | 2 | 4 | 1800 | 2 |
| 1 | 185 | 2 | 0 | 2 | 1 | 1230 | 4 |
| 1 | 235 | 2 | 0 | 1 | 4 | 1000 | 2 |
| 2 | 210 | 1 | 0 | 2 | 1 | 2000 | 5 |
| 2 | 73 | 1 | 0 | 1 | 1 | 400 | 8 |
| 2 | 30 | 1 | 0 | 1 | 3 | 100 | 2 |
| 2 | 100 | 1 | 0 | 1 | 1 | 450 | 2 |
| 2 | 195 | 1 | 0 | 2 | 1 | 1150 | 8 |
| 1 | 180 | 2 | 0 | 2 | 2 | 1470 | 29 |
| 1 | 85 | 2 | 0 | 2 | 1 | 100 | 47 |
| 1 | 140 | 2 | 0 | 2 | 1 | 850 | 45 |
| 1 | 635 | 2 | 1 | 2 | 1 | 5400 | 11 |
| 1 | 80 | 2 | 0 | 2 | 1 | 500 | 33 |
| 1 | 210 | 2 | 0 | 2 | 4 | 1450 | 7 |
| 1 | 125 | 2 | 0 | 2 | 1 | 1400 | 2 |
| 1 | 60 | 2 | 0 | 1 | 1 | 800 | 4 |
| 1 | 80 | 2 | 0 | 2 | 1 | 650 | 4 |
| 1 | 205 | 2 | 0 | 2 | 4 | 1250 | 4 |
| 1 | 155 | 2 | 0 | 2 | 1 | 450 | 2 |
| 1 | 135 | 2 | 0 | 2 | 1 | 550 | 20 |
| 1 | 225 | 2 | 0 | 2 | 4 | 2100 | 9 |
| 1 | 145 | 2 | 0 | 1 | 1 | 900 | 5 |
| 1 | 265 | 2 | 0 | 2 | 4 | 3100 | 4 |
| 1 | 65 | 1 | 0 | 2 | 1 | 500 | 2 |
| 1 | 120 | 2 | 0 | 1 | 1 | 400 | 2 |
| 1 | 145 | 2 | 0 | 2 | 1 | 800 | 4 |
| 1 | 70 | 2 | 0 | 2 | 4 | 450 | 5 |
| 1 | 105 | 2 | 0 | 2 | 1 | 700 | 12 |
| 1 | 195 | 2 | 0 | 2 | 1 | 550 | 4 |
| 1 | 150 | 2 | 0 | 1 | 1 | 1185 | 3 |
| 1 | 110 | 2 | 0 | 2 | 1 | 650 | 4 |
| 1 | 60 | 2 | 0 | 2 | 4 | 500 | 3 |
| 1 | 210 | 2 | 0 | 1 | 4 | 2550 | 8 |
| 2 | 155 | 1 | 0 | 2 | 1 | 1600 | 7 |
| 2 | 45 | 1 | 0 | 2 | 1 | 250 | 3 |
| 2 | 70 | 1 | 0 | 2 | 1 | 450 | 2 |
| 2 | 50 | 1 | 0 | 2 | 1 | 90 | 15 |
| 2 | 15 | 1 | 0 | 2 | 1 | 350 | 1 |
| 2 | 220 | 2 | 0 | 2 | 1 | 1350 | 4 |
| 2 | 145 | 2 | 0 | 2 | 1 | 800 | 30 |
| 2 | 100 | 2 | 0 | 2 | 2 | 870 | 5 |
| 2 | 195 | 2 | 0 | 2 | 1 | 1500 | 9 |
| 2 | 70 | 2 | 0 | 2 | 2 | 450 | 65 |
| 2 | 80 | 1 | 0 | 2 | 1 | 250 | 8 |
| 2 | 85 | 1 | 0 | 2 | 1 | 400 | 6 |

| | | | | | | | |
|---|---|---|---|---|---|---|---|
| 2 | 75 | 1 | 0 | 2 | 1 | 450 | 3 |
| 2 | 65 | 1 | 0 | 1 | 1 | 400 | 3 |
| 2 | 60 | 1 | 0 | 2 | 1 | 320 | 2 |
| 1 | 330 | 2 | 0 | 2 | 1 | 5000 | 14 |
| 1 | 305 | 2 | 0 | 1 | 4 | 4600 | 11 |
| 1 | 110 | 2 | 0 | 1 | 1 | 430 | 4 |
| 1 | 105 | 2 | 0 | 2 | 1 | 700 | 2 |
| 1 | 135 | 2 | 0 | 2 | 1 | 350 | 6 |
| 1 | 120 | 2 | 0 | 2 | 1 | 500 | 4 |
| 1 | 70 | 2 | 0 | 2 | 2 | 1100 | 3 |
| 1 | 245 | 2 | 0 | 2 | 2 | 1850 | 12 |
| 1 | 70 | 2 | 0 | 2 | 1 | 350 | 3 |
| 1 | 100 | 2 | 0 | 1 | 1 | 750 | 4 |
| 1 | 165 | 2 | 0 | 2 | 1 | 500 | 5 |
| 1 | 185 | 2 | 0 | 2 | 1 | 400 | 4 |
| 1 | 170 | 2 | 0 | 1 | 3 | 1700 | 4 |
| 1 | 95 | 2 | 0 | 2 | 1 | 500 | 6 |
| 1 | 175 | 2 | 0 | 2 | 1 | 900 | 3 |
| 2 | 130 | 2 | 0 | 2 | 1 | 300 | 8 |
| 2 | 40 | 2 | 0 | 1 | 3 | 250 | 2 |
| 2 | 55 | 2 | 0 | 2 | 1 | 350 | 2 |
| 2 | 45 | 2 | 0 | 1 | 3 | 50 | 2 |
| 2 | 55 | 2 | 0 | 1 | 1 | | 1 |
| 1 | 145 | 2 | 0 | 1 | 1 | 1300 | 6 |
| 1 | 185 | 2 | 0 | 2 | 1 | 1300 | 2 |
| 1 | 55 | 1 | 0 | 1 | 1 | 600 | 2 |
| 1 | 235 | 2 | 0 | 2 | 1 | 250 | 5 |
| 1 | 115 | 2 | 0 | 2 | 4 | 1300 | 3 |
| 2 | 65 | 2 | 0 | 2 | 1 | 500 | 2 |
| 2 | 185 | 2 | 0 | 1 | 2 | 2050 | 8 |
| 2 | 45 | 2 | 0 | 2 | 3 | 100 | 2 |
| 2 | 55 | 2 | 0 | 2 | 2 | 300 | 3 |
| 2 | 30 | 2 | 0 | 1 | 1 | 200 | 15 |
| 2 | 105 | 1 | 0 | 1 | 1 | 800 | 3 |
| 2 | 55 | 1 | 0 | 2 | 1 | 250 | 11 |
| 2 | 70 | 1 | 0 | 2 | 1 | 310 | 16 |
| 2 | 95 | 1 | 0 | 2 | 2 | 200 | 6 |
| 2 | 75 | 1 | 0 | 1 | 2 | 300 | 5 |
| 2 | 110 | 1 | 0 | 1 | 1 | 600 | 7 |
| 2 | 135 | 1 | 0 | 2 | 2 | 600 | 3 |
| 2 | 100 | 1 | 0 | 2 | 1 | 350 | 2 |
| 2 | 130 | 1 | 0 | 2 | 1 | 560 | 1 |
| 2 | 200 | 1 | 0 | 1 | 1 | 400 | 7 |
| 2 | 30 | 1 | 0 | 2 | 3 | 300 | 1 |
| 2 | 35 | 1 | 0 | 1 | 3 | 250 | 2 |
| 2 | 65 | 1 | 0 | 2 | 1 | 350 | 1 |
| 2 | 125 | 1 | 0 | 2 | 1 | 300 | 4 |
| 2 | 140 | 1 | 0 | 1 | 1 | 1400 | 13 |
| 1 | 105 | 1 | 1 | 2 | 1 | 450 | 7 |
| 1 | 155 | 2 | 0 | 2 | 4 | 700 | 5 |
| 1 | 100 | 2 | 0 | 1 | 1 | 400 | 4 |
| 1 | 600 | 2 | 0 | 1 | 4 | 4850 | 12 |
| 1 | 250 | 2 | 0 | 2 | 1 | 2650 | 12 |
| 2 | 70 | 1 | 0 | 1 | 1 | 200 | 1 |
| 2 | 45 | 1 | 0 | 2 | 1 | 350 | 2 |
| 2 | 70 | 1 | 0 | 1 | 1 | 300 | 3 |
| 2 | 90 | 1 | 0 | 2 | 1 | 350 | 2 |

| | | | | | | | |
|---|---|---|---|---|---|---|---|
| 2 | 175 | 1 | 0 | 2 | 1 | 1300 | 6 |
| 1 | 110 | 1 | 1 | 2 | 1 | 700 | 39 |
| 1 | 155 | 2 | 1 | 2 | 1 | 100 | 42 |
| 1 | 100 | 2 | 0 | 2 | 1 | 500 | 4 |
| 1 | 185 | 2 | 0 | 2 | 4 | 1250 | 3 |
| 1 | 145 | 2 | 0 | 2 | 1 | 520 | 5 |
| 1 | 370 | 2 | 0 | 2 | 4 | 1350 | 18 |
| 1 | 260 | 2 | 0 | 2 | 4 | 4240 | 6 |
| 1 | 550 | 2 | 0 | 1 | 2 | 4900 | 8 |
| 1 | 190 | 2 | 0 | 1 | 1 | 1300 | 4 |
| 1 | 140 | 2 | 0 | 1 | 1 | 1400 | 20 |
| 2 | 30 | 1 | 0 | 2 | 3 | 50 | 2 |
| 2 | 30 | 1 | 0 | 2 | 3 | 300 | 3 |
| 2 | 75 | 1 | 0 | 1 | 3 | 450 | 3 |
| 2 | 50 | 1 | 0 | 2 | 3 | 150 | 2 |
| 2 | 35 | 1 | 0 | 1 | 3 | 100 | 8 |
| 2 | 110 | 1 | 0 | 2 | 1 | 500 | 13 |
| 2 | 70 | 1 | 0 | 1 | 1 | 600 | 7 |
| 2 | 180 | 1 | 0 | 2 | 1 | 1400 | 6 |
| 2 | 55 | 1 | 0 | 2 | 1 | 200 | 12 |
| 2 | 155 | 1 | 0 | 2 | 1 | 1500 | 6 |
| 1 | 250 | 2 | 0 | 1 | 1 | 1770 | 8 |
| 1 | 115 | 2 | 0 | 2 | 4 | 1000 | 6 |
| 1 | 195 | 2 | 0 | 2 | 4 | 2300 | 8 |
| 1 | 225 | 2 | 0 | 2 | 4 | 1800 | 9 |
| 1 | 185 | 2 | 0 | 2 | 4 | 2000 | 5 |
| 2 | 30 | 1 | 0 | 2 | 3 | 200 | 4 |
| 2 | 60 | 1 | 1 | 2 | 2 | 350 | 4 |
| 2 | 40 | 1 | 0 | 2 | 3 | 100 | 2 |
| 2 | 110 | 1 | 0 | 2 | 1 | 480 | 8 |
| 2 | 65 | 1 | 0 | 1 | 3 | 550 | 2 |
| 2 | 60 | 1 | 0 | 2 | 1 | 450 | 3 |
| 2 | 35 | 1 | 0 | 1 | 3 | 300 | 1 |
| 2 | 35 | 1 | 0 | 2 | 3 | 250 | 1 |
| 2 | 80 | 1 | 0 | 2 | 1 | 350 | 2 |
| 2 | 65 | 1 | 0 | 2 | 1 | 250 | 6 |
| 2 | 60 | 2 | 0 | 2 | 1 | 350 | 13 |
| 2 | 55 | 2 | 0 | 2 | 1 | 500 | 17 |
| 2 | 50 | 2 | 0 | 2 | 1 | 200 | 1 |
| 2 | 160 | 2 | 0 | 2 | 1 | 550 | 8 |
| 2 | 65 | 2 | 0 | 1 | 2 | 450 | 2 |
| 2 | 135 | 2 | 0 | 2 | 2 | 450 | 2 |
| 2 | 55 | 2 | 0 | 2 | 1 | 250 | 3 |
| 2 | 45 | 2 | 0 | 1 | 3 | 400 | 1 |
| 2 | 60 | 2 | 0 | 1 | 3 | 300 | 2 |
| 2 | 60 | 2 | 0 | 2 | 1 | 200 | 1 |
