# Supplementary material for: Incidence and risk factors for postoperative lingual neuropraxia following airway instrumentation: A retrospective matched case-control study
Source: PLoS One. 2018 Jan 12;13(1):e0190589. doi: 10.1371/journal.pone.0190589 (PMC5766107; doi:10.1371/journal.pone.0190589)
Supplement: S3 Table — (PDF) [file pone.0190589.s003.pdf]

Variable	Description	Coding					
		0	1	2	3	4	Remark
Group	To distinguish whether a patient occurred postoperative lingual neuropraxia	No	Yes				
No	Match pair id						
Gender	Gender		Male	Female			
Age	Patient's age on OP date						
Age_Group	Age Group(divided into 4 groups)		≦30 y/o	30-50 y/o	50-70 y/o	>70 y/o	
Height	Paitent's height(cm)						
Weight	Paitent's weight(kg)						
BMI	Paitent's BMI						
BMI_Group	BMI Group(divided into 3 groups)		Normal	Underweight	Overweight or Obese		
OP_site	Surgery region	Not head & neck	Head & neck				
ASA	ASA		1	2	>=3		
ASA_Type	Anesthesia Type		ETGA	LMA			
ASA_time	Duration of anesthesia(min.)						
Size_Group	Size of airway device		Usual	Large			
Difficulty	Difficult intubation	No	Yes				
Experience	Experience of operator		Junior	Senior			
Posture	Intraoperative positioning		Supine	Lateral decubitus	Lithotomy	Prone	
Fluid	Intraoperative fluid(ml)						
LOS	Length of hospital stay(days)						
